# 3,8-Dimethyl­quinazoline-2,4(1*H*,3*H*)-dione

**DOI:** 10.1107/S1600536811025232

**Published:** 2011-07-06

**Authors:** Wei-Yan Qin, Bo Liu, Cong-Wen Duan, Qing-Xiu Yin

**Affiliations:** a Key Laboratory of Green Chemical Technology, College of Heilongjiang Province, School of Chemistry and Environmental Engineering, Harbin University of Science and Technology, Harbin 150040, People’s Republic of China

## Abstract

In the title compound, C_10_H_10_N_2_O_2_, all non-H atoms are approximately co-planar with an r.m.s. deviation of 0.016 Å. In the crystal, mol­ecules are linked into inversion dimers by pairs of N—H⋯O hydrogen bonds. Chains along [010] are buiilt up by π–π inter­actions [centroid–centroid distance = 3.602 (1) Å] between the benzene and piperazine rings of adjacent mol­ecules.

## Related literature

For the synthesis and background to the title compound, see Feng *et al.* (2010 ▶).
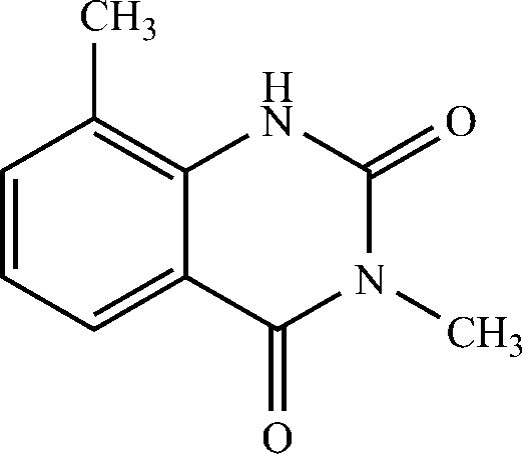


## Experimental

### 

#### Crystal data

C_10_H_10_N_2_O_2_
*M* *_r_* = 190.20
Monoclinic, 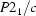

*a* = 8.3604 (17) Å
*b* = 4.8599 (10) Å
*c* = 22.288 (5) Å
β = 92.09 (3)°
*V* = 905.0 (3) Å^3^
*Z* = 4
Mo *K*α radiation
μ = 0.10 mm^−1^
*T* = 295 K
0.29 × 0.23 × 0.19 mm

#### Data collection

Rigaku R-AXIS RAPID diffractometer
Absorption correction: multi-scan (*ABSCOR*; Higashi, 1995 ▶) *T*_min_ = 0.972, *T*_max_ = 0.981
8373 measured reflections
2085 independent reflections
1497 reflections with *I* > 2σ(*I*)
*R*_int_ = 0.030

#### Refinement

*R*[*F* ^2^ > 2σ(*F* ^2^)] = 0.043
*wR*(*F* ^2^) = 0.143
*S* = 1.10
2085 reflections
133 parameters
H atoms treated by a mixture of independent and constrained refinement
Δρ_max_ = 0.22 e Å^−3^
Δρ_min_ = −0.18 e Å^−3^

### 

Data collection: *RAPID-AUTO* (Rigaku, 1998 ▶); cell refinement: *RAPID-AUTO*; data reduction: *CrystalStructure* (Rigaku/MSC, 2002) ▶; program(s) used to solve structure: *SHELXS97* (Sheldrick, 2008 ▶); program(s) used to refine structure: *SHELXL97* (Sheldrick, 2008 ▶); molecular graphics: *SHELXTL* (Sheldrick, 2008 ▶); software used to prepare material for publication: *SHELXL97*.

## Supplementary Material

Crystal structure: contains datablock(s) I, global. DOI: 10.1107/S1600536811025232/ng5192sup1.cif

Structure factors: contains datablock(s) I. DOI: 10.1107/S1600536811025232/ng5192Isup2.hkl

Supplementary material file. DOI: 10.1107/S1600536811025232/ng5192Isup3.cml

Additional supplementary materials:  crystallographic information; 3D view; checkCIF report

## Figures and Tables

**Table 1:** Hydrogen-bond geometry (Å, °)

*D*—H⋯*A*	*D*—H	H⋯*A*	*D*⋯*A*	*D*—H⋯*A*
N2—H1⋯O2^i^	0.888 (19)	2.011 (19)	2.8931 (17)	171.8 (17)

Symmetry code: (i) 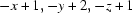.

**Fig. 1.**
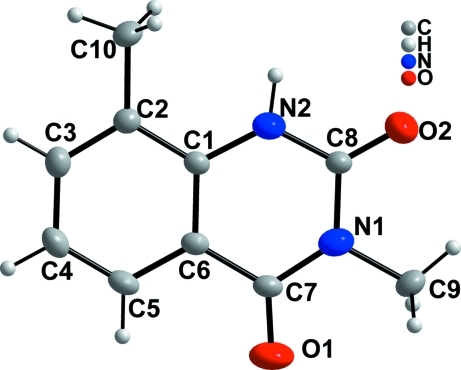
The molecular structure of the title compound, showing displacement ellipsoids at the 50% probability level for non-H atoms.

**Fig. 2.**
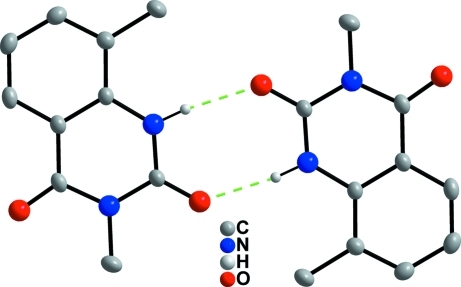
A dimer structure showing the N—H···O hydrogen bonds, no involving H atoms have been omitted for clarity.

**Fig. 3.**
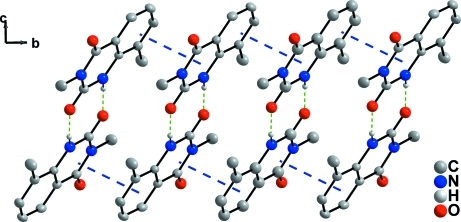
A partial packing view, showing chain structure forming by π-π interactions along [010] direction, no involving H atoms have been omitted for clarity.

## supplementary crystallographic information

### Comment

The title compound is the intermediate of a kind of highly potent and selective insecticide (Feng *et al.*, 2010). Herein, we report the synthesis and crystal structure of the title compound.

In the title compound, C_10_H_10_N_2_O_2_, all non-hydrogen atoms lie on the same plane with the Rms about 0.016 Å, the largest deviation being 0.037 (1) Å for atom O2 (Figure 1).

The isolated title compound molecules are linked by N—H···O hydrogen bonds into dimers (Figure 2, Table 1). Furthermore, the chain structures alone [010] direction are bulit up by /pi-/pi interatcions (center to center distances of 3.602 (1) Å) between the phenyl groups and piperazinyl groups of the adjacent molecules (Figure 3).

### Experimental

The title compound was synthesized as the reference method (Feng *et al.*, 2010): To a solution of 2-amino-*N*,3-dimethylbenzamide (1.64 g, 1.0 mmol) in THF (20 ml), bis(trichloromethyl)-carbonate (1.0 g, 0.33 mmol) was added, and then keep stirring for 2h. After that THF was removed and water (20 ml) was added slowly. The resulting suspension was filtered, and the solids were washed with water (15 ml) and dried (yield 65%). The crystals suitable for X-ray diffraction were obtained by slow evaporation from methanol solution at room temperature for several days.

### Refinement

H atoms bound to C atoms were placed in calculated positions and treated as riding on their parent atoms, with C—H = 0.93 Å (aromatic); C—H = 0.96 Å (methyl), and with *U*_iso_(H) = 1.2*U*_eq_(C), while N-bound H atom was found from Fourier-map and was freely refined.

### Crystal data

**Table:**

C_10_H_10_N_2_O_2_	*F*(000) = 400
*M**_r_* = 190.20	*D*_x_ = 1.396 Mg m^−^^3^
Monoclinic, *P*2_1_/*c*	Mo *K*α radiation, λ = 0.71073 Å
Hall symbol: -P 2ybc	Cell parameters from 6086 reflections
*a* = 8.3604 (17) Å	θ = 3.0–27.5°
*b* = 4.8599 (10) Å	µ = 0.10 mm^−^^1^
*c* = 22.288 (5) Å	*T* = 295 K
β = 92.09 (3)°	Block, colorless
*V* = 905.0 (3) Å^3^	0.29 × 0.23 × 0.19 mm
*Z* = 4	

### Data collection

**Table:**

Rigaku R-AXIS RAPID diffractometer	2085 independent reflections
Radiation source: fine-focus sealed tube	1497 reflections with *I* > 2σ(*I*)
graphite	*R*_int_ = 0.030
ω scan	θ_max_ = 27.5°, θ_min_ = 3.0°
Absorption correction: multi-scan (*ABSCOR*; Higashi, 1995)	*h* = −10→10
*T*_min_ = 0.972, *T*_max_ = 0.981	*k* = −6→6
8373 measured reflections	*l* = −28→28

### Refinement

**Table:**

Refinement on *F*^2^	Primary atom site location: structure-invariant direct methods
Least-squares matrix: full	Secondary atom site location: difference Fourier map
*R*[*F*^2^ > 2σ(*F*^2^)] = 0.043	Hydrogen site location: inferred from neighbouring sites
*wR*(*F*^2^) = 0.143	H atoms treated by a mixture of independent and constrained refinement
*S* = 1.10	*w* = 1/[σ^2^(*F*_o_^2^) + (0.0833*P*)^2^ + 0.0221*P*] where *P* = (*F*_o_^2^ + 2*F*_c_^2^)/3
2085 reflections	(Δ/σ)_max_ < 0.001
133 parameters	Δρ_max_ = 0.22 e Å^−^^3^
0 restraints	Δρ_min_ = −0.18 e Å^−^^3^

### Special details

**Table:**

Geometry. All esds (except the esd in the dihedral angle between two l.s. planes) are estimated using the full covariance matrix. The cell esds are taken into account individually in the estimation of esds in distances, angles and torsion angles; correlations between esds in cell parameters are only used when they are defined by crystal symmetry. An approximate (isotropic) treatment of cell esds is used for estimating esds involving l.s. planes.
Refinement. Refinement of F^2^ against ALL reflections. The weighted R-factor wR and goodness of fit S are based on F^2^, conventional R-factors R are based on F, with F set to zero for negative F^2^. The threshold expression of F^2^ > 2sigma(F^2^) is used only for calculating R-factors(gt) etc. and is not relevant to the choice of reflections for refinement. R-factors based on F^2^ are statistically about twice as large as those based on F, and R- factors based on ALL data will be even larger.

### Fractional atomic coordinates and isotropic or equivalent isotropic displacement parameters (Å^2^)

**Table:**

	*x*	*y*	*z*	*U*_iso_*/*U*_eq_	
C1	0.36317 (16)	0.6713 (2)	0.37609 (6)	0.0319 (3)	
C2	0.47592 (17)	0.4802 (3)	0.35641 (6)	0.0378 (3)	
C3	0.43348 (19)	0.3256 (3)	0.30632 (7)	0.0436 (4)	
H3	0.5058	0.1965	0.2927	0.052*	
C4	0.2866 (2)	0.3552 (3)	0.27525 (7)	0.0459 (4)	
H4	0.2625	0.2481	0.2415	0.055*	
C5	0.17879 (18)	0.5421 (3)	0.29463 (6)	0.0420 (4)	
H5	0.0807	0.5633	0.2741	0.050*	
C6	0.21521 (16)	0.7020 (3)	0.34530 (6)	0.0343 (3)	
C7	0.09745 (16)	0.8962 (3)	0.36722 (6)	0.0371 (3)	
C8	0.29243 (17)	1.0222 (3)	0.44833 (6)	0.0359 (3)	
C9	0.03023 (19)	1.2458 (4)	0.44098 (7)	0.0499 (4)	
H9A	0.0786	1.3429	0.4745	0.075*	
H9B	−0.0632	1.1502	0.4536	0.075*	
H9C	0.0002	1.3743	0.4099	0.075*	
C10	0.63640 (19)	0.4454 (4)	0.38882 (8)	0.0536 (4)	
H10A	0.6956	0.3027	0.3698	0.080*	
H10B	0.6206	0.3963	0.4299	0.080*	
H10C	0.6950	0.6150	0.3874	0.080*	
N1	0.14501 (13)	1.0469 (2)	0.41799 (5)	0.0374 (3)	
H1	0.486 (2)	0.829 (3)	0.4482 (8)	0.056 (5)*	
N2	0.39514 (14)	0.8334 (2)	0.42650 (5)	0.0368 (3)	
O1	−0.03642 (13)	0.9286 (3)	0.34450 (5)	0.0551 (4)	
O2	0.32575 (13)	1.1631 (2)	0.49285 (5)	0.0510 (3)	

### Atomic displacement parameters (Å^2^)

**Table:**

	*U*^11^	*U*^22^	*U*^33^	*U*^12^	*U*^13^	*U*^23^
C1	0.0322 (7)	0.0340 (6)	0.0292 (6)	−0.0020 (5)	−0.0025 (5)	0.0018 (5)
C2	0.0353 (7)	0.0401 (7)	0.0378 (7)	0.0022 (6)	0.0001 (6)	0.0014 (6)
C3	0.0483 (9)	0.0425 (8)	0.0404 (8)	0.0021 (7)	0.0063 (6)	−0.0053 (6)
C4	0.0548 (9)	0.0472 (8)	0.0354 (7)	−0.0065 (7)	−0.0019 (7)	−0.0077 (6)
C5	0.0393 (8)	0.0504 (8)	0.0355 (7)	−0.0073 (7)	−0.0088 (6)	0.0019 (6)
C6	0.0329 (7)	0.0372 (7)	0.0324 (7)	−0.0013 (6)	−0.0040 (5)	0.0042 (5)
C7	0.0321 (7)	0.0425 (7)	0.0361 (7)	−0.0007 (6)	−0.0069 (5)	0.0052 (6)
C8	0.0321 (7)	0.0410 (7)	0.0343 (7)	0.0042 (6)	−0.0046 (5)	−0.0005 (6)
C9	0.0401 (8)	0.0572 (9)	0.0522 (9)	0.0160 (7)	0.0007 (7)	−0.0022 (8)
C10	0.0378 (8)	0.0624 (10)	0.0599 (10)	0.0144 (8)	−0.0063 (7)	−0.0102 (8)
N1	0.0291 (6)	0.0434 (6)	0.0393 (6)	0.0069 (5)	−0.0045 (5)	0.0007 (5)
N2	0.0306 (6)	0.0436 (6)	0.0356 (6)	0.0068 (5)	−0.0081 (5)	−0.0050 (5)
O1	0.0362 (6)	0.0710 (8)	0.0566 (7)	0.0086 (5)	−0.0182 (5)	−0.0021 (6)
O2	0.0438 (6)	0.0614 (7)	0.0467 (6)	0.0140 (5)	−0.0139 (5)	−0.0198 (5)

### Geometric parameters (Å, °)

**Table:**

C1—N2	1.3901 (17)	C7—N1	1.3938 (17)
C1—C6	1.4005 (18)	C8—O2	1.2291 (17)
C1—C2	1.4049 (19)	C8—N2	1.3588 (18)
C2—C3	1.381 (2)	C8—N1	1.3891 (17)
C2—C10	1.5099 (19)	C9—N1	1.4675 (19)
C3—C4	1.395 (2)	C9—H9A	0.9600
C3—H3	0.9300	C9—H9B	0.9600
C4—C5	1.361 (2)	C9—H9C	0.9600
C4—H4	0.9300	C10—H10A	0.9600
C5—C6	1.395 (2)	C10—H10B	0.9600
C5—H5	0.9300	C10—H10C	0.9600
C6—C7	1.461 (2)	N2—H1	0.888 (19)
C7—O1	1.2217 (16)		
			
N2—C1—C6	118.44 (12)	O2—C8—N2	122.52 (12)
N2—C1—C2	121.04 (11)	O2—C8—N1	121.05 (13)
C6—C1—C2	120.52 (12)	N2—C8—N1	116.43 (11)
C3—C2—C1	117.16 (13)	N1—C9—H9A	109.5
C3—C2—C10	121.54 (13)	N1—C9—H9B	109.5
C1—C2—C10	121.29 (12)	H9A—C9—H9B	109.5
C2—C3—C4	122.73 (14)	N1—C9—H9C	109.5
C2—C3—H3	118.6	H9A—C9—H9C	109.5
C4—C3—H3	118.6	H9B—C9—H9C	109.5
C5—C4—C3	119.49 (13)	C2—C10—H10A	109.5
C5—C4—H4	120.3	C2—C10—H10B	109.5
C3—C4—H4	120.3	H10A—C10—H10B	109.5
C4—C5—C6	120.07 (13)	C2—C10—H10C	109.5
C4—C5—H5	120.0	H10A—C10—H10C	109.5
C6—C5—H5	120.0	H10B—C10—H10C	109.5
C5—C6—C1	120.02 (13)	C8—N1—C7	124.86 (11)
C5—C6—C7	120.04 (12)	C8—N1—C9	117.83 (12)
C1—C6—C7	119.92 (12)	C7—N1—C9	117.31 (11)
O1—C7—N1	119.92 (13)	C8—N2—C1	124.52 (11)
O1—C7—C6	124.26 (13)	C8—N2—H1	111.4 (11)
N1—C7—C6	115.81 (11)	C1—N2—H1	124.1 (11)

### Hydrogen-bond geometry (Å, °)

**Table:**

*D*—H···*A*	*D*—H	H···*A*	*D*···*A*	*D*—H···*A*
N2—H1···O2^i^	0.888 (19)	2.011 (19)	2.8931 (17)	171.8 (17)

Symmetry codes: (i) −*x*+1, −*y*+2, −*z*+1.
